# Gamma Irradiation of Fluorocarbon Polymers*

**DOI:** 10.6028/jres.065A.038

**Published:** 1961-08-01

**Authors:** Roland E. Florin, Leo A. Wall

## Abstract

Several fluorocarbon polymers were irradiated with Co^60^ gamma radiation at doses up to 10^22^ ev/g. The polymers studied included polytetrafluoroethylene, polytrifluoroethylene, polychlorotrifluoroethylene, a copolymer of tetrafluoroethylene with hexafluoropropylene, and several rubbery vinylidene fluoride copolymers. *G*-values were measured for volatile products, for free radicals detected by electron spin resonance, and, in the case of polychlorotrifluoroethylene, for scissions. The course of degradation or crosslinking was followed by zero-strength-time and tensile-strength measurements. It was found that for polytetrafluoroethylene and its hexafluoropropylene copolymer the presence of air-accelerated scission drastically. The mechanism of the radiation-induced changes is discussed in terms of free-radical intermediates.

## 1. Introduction

In spite of their outstanding chemical and thermal stability, fluorocarbon polymers are usually classed among the poorest in resistance to radiation. They are considered to undergo degradation exclusively, and this degradation produces corrosive products [1–6].^1^ If we include materials having some hydrocarbon groups, such as perfluoroalkyl-substituted silicones, hexafluorobutyl acrylate, and vinylidene fluoride copolymers, there is, however, a variation in behavior; for example, cross linking can occur [1]. The radiation dose at which most useful properties are lost ranges from a few megaroentgens for polytetrafluoroethylene to over 100 Mr for hexafluoropropylenevinylidene fluoride copolymers.

Aside from the striking contrast between the radiation resistance and the chemical and thermal resistance of these polymers, there are, however, other reasons for questioning the implication of extreme radiation sensitivity. An initial increase in impact strength of polytetrafluoroethylene was reported to take place prior to deterioration [3], and tensile strengths of 50 percent were retained under some circumstances [8] after 50 Mr of radiation. Most practical evaluations are made in the presence of air and moisture at 25 °C; results in vacuum can differ profoundly from these in some instances. Small fluorocarbon molecules studied in sealed containers [9,10] have been found to be more stable towards radiation when air is absent. Because of the influence of diffusion (of oxygen inward and degradation products outward) the observed effects may depend upon the sample dimensions. Although radiation resistance approaching that of butadiene-styrene rubbers, marginally usable after a dosage of 10^3^ Mr, is hardly to be expected of fluorocarbon materials, they may be superior in special combinations of dose, temperature, and environment.

More knowledge of the chemical mechanism of the radiation-induced changes was sought in this work by a study of volatile end products, intermediate radicals, and mechanical and flow properties related to molecular weight. Mass spectrometry and electron spin resonance (ESR) appeared adaptable for the first two. The study of molecular weight and cross linking would ordinarily be best conducted by the conventional methods of light scattering, solution viscosity, or swelling. However, since the measurement of any solution property of polytetrafluoroethylene offers extraordinary difficulties and the basic relations with molecular weight have not yet been established for most other fluorocarbon polymers, most reliance in this study was placed upon the semiquantitative indications furnished by tensile strength and zero-strength-time (ZST) determinations.

## 2. Experimental Procedure

The polymers studied were:

1. PTFE (Polytetrafluoroethylene)
2. TFE-HFP (Copolymer of tetrafluoroethylene and hexafluoropropylene)
3. PCTFE (Polychlorotrifluoroethylene)
4. PTrFE (Polytrifluoroethylene)
5. CTFE-VF (Copolymer of chlorotrifluoroethylene and vinylidene fluoride)
6. HFP-VF (Copolymer of hexafluoropropylene and vinylidene fluoride)
7. PTFS (Poly-*α*, *β, β*-trifluorostyrene)
8. PPFS (Poly-2,3,4,5,6-pentafluorostyrene)

Most of these polymers were supplied commercially; however, PTrFE was prepared in the laboratory in an aqueous persulfate system at 60 to 80 °C, PPFS was prepared in the laboratory, and the PTFS was supplied by R. S. Corley of Polaroid Corp. Available analytical data on the copolymers are shown in table 1.

The radiation facility was a 2,000-curie Co^60^ source having an exposure dose rate near 0.5×10^6^ R/hr. Methods for calculating the absorbed dose have been described [10]. Doses were in the range 1 to 200 × 10^6^ R, and irradiations were made usually at a temperature of 20±2 °C.

For observations of volatiles, about 0.1 g of the polymer was used in powered form, if possible, in an evacuated hard glass tube lined with foil of aluminum, silver, or nickel. Tubes were evacuated to pressures less than 10^−4^ mm of Hg before being sealed off. There was usually a delay of weeks to months before examination by mass spectrometer; thus any post-irradiation effects had generally taken place before the analysis was made. However, the effect of post-irradiation heating was studied for PTFE. The samples for zero-strength-time tests (ZST) [11, 12] were ordinarily pressed from molding powder, at the specified time and temperature, to the standard thickness and cut to usual size and notched shape. The ZST specimens of TFE-HFP copolymer were cut from commercial sheets of 0.060 in. and 0.040 in. thicknesses. Specimens were sealed in glass tubes, either in vacuum or in air, for the irradiation. The irradiated specimens were opened immediately before testing. Two to five replicate specimens were included in each tube. The conditions for molding and for the ZST determination are shown in table 2. Some specimens irradiated in air, rapidly became too fragile to handle; in other cases, supplementary ZST determinations were made upon weaker specimens at full cross section without notches.

Samples for ESR measurements were usually cut in the form of a movable plug, sealed in 5-mm glass tubes after many hours of evacuation, and observed after briefly heating one end of the irradiated container to remove the signal due to glass, while cooling the other end with liquid nitrogen. PTFE samples were heated during evacuation, in some instances to 400 °C. Powdered or rubbery samples or those to be observed at very low temperature, were sealed in thin-walled tubes of Corning No. 7943 fused silica, a special high-purity grade prepared by a vapor-phase process. The signal from irradiated containers of this material is sharp and narrow, and its interference can often be ignored or corrected for. ESR observations were made with a Varian 4500 instrument at frequencies in the neighborhood of 9,000 to 9,600 Mc and fields in the neighborhood of 3,300 gauss. Rectangular cavities operating in the TE 012 mode were used; for low temperatures the cavity had a hole nearly 10 mm in diam and accommodated a Dewar-walled tube carrying a stream of cold nitrogen. Quantitative estimates were made by double integration of the first-derivative curves and comparison with those obtained with copper sulfate pentahydrate or diphenyl picryl hydrazyl.

## 3. Results

The *G*-values, in molecules per 100 ev, of the volatile products from irradiation of the polymers are shown in several tables: PTFE in table 3; copolymer TFE-HFP in table 4; PCTFE in table 5; PTrFE in table 6; and copolymer HFP-VF in table 7. All irradiations in these tables were made at 20±2 °C in vacuum.

**Figure 1:**
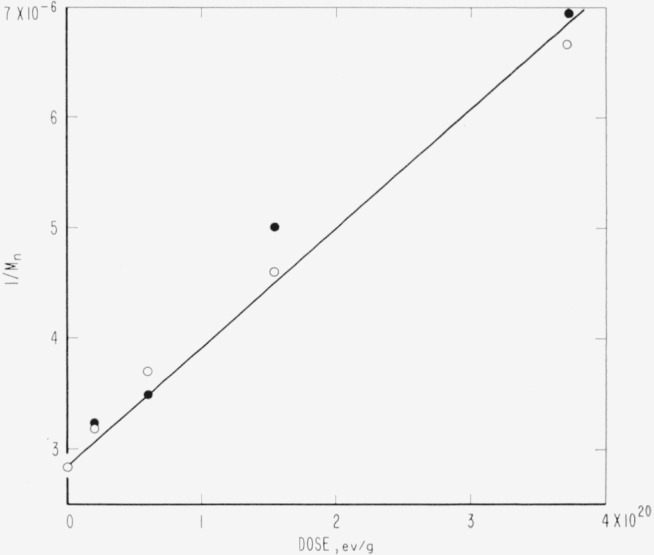
Loss of molecular weight of polychlorotrifluoroethylene during irradiation. ○, irradiated in vacuum. ●, irradiated in air.

**Figure 2:**
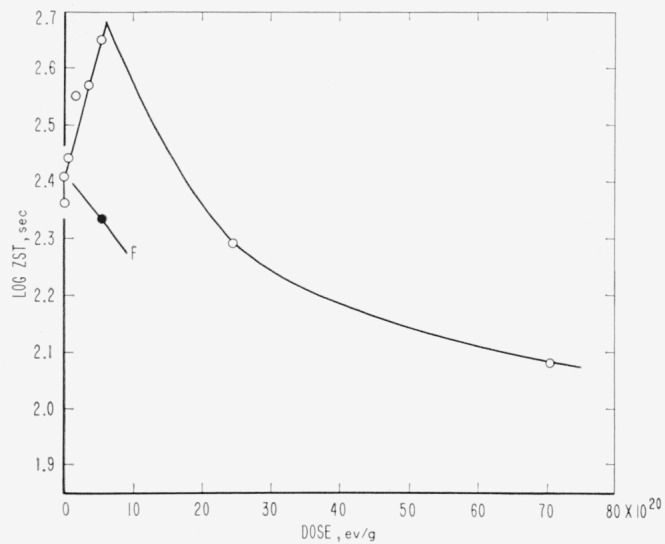
Zero-strength-time of irradiated copolymer tetrafluoroethylene-hexafluoropropylene. ○, irradiated in vacuum. ●, irradiated in air. F, too weak to handle after 24×10^20^ev/g.

**Figure 3:**
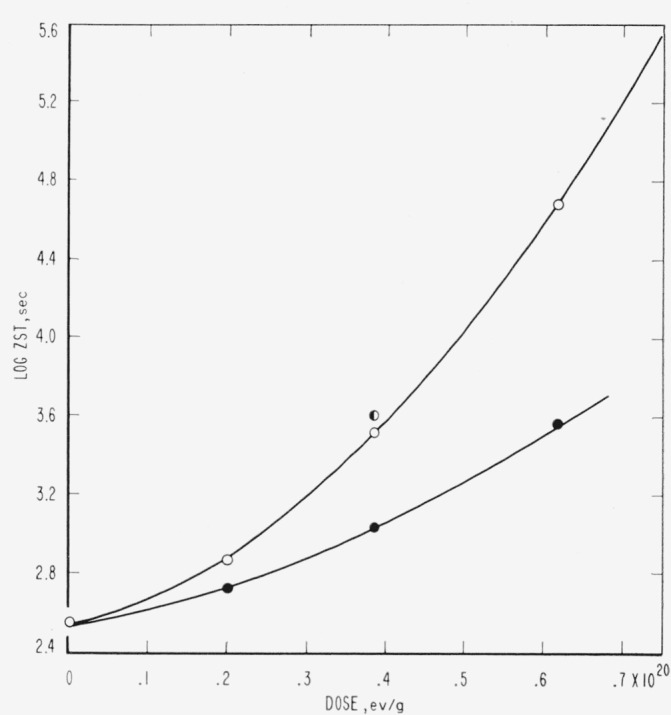
Zero-strength-time of irradiated copolymer hexafluoropropylene-vinylidene fluoride. ○, irradiated in vacuum. ◐, irradiated in vacuum, postheated 100 °C, 30 min. ●, irradiated in air. Log ZST greater than 5.4 at doses of 5.7 and 74×10^20^ ev/g in vacuum.

**Figure 4:**
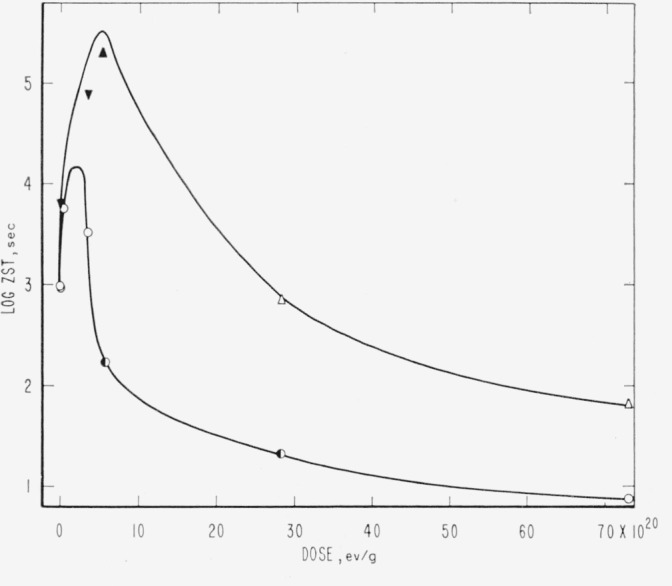
Zero-strength-time of irradiated copolymers chlorotriflurorethylene-vinylidene fluoride. ○, high chlorine content in air. ◐, high chlorine content in vacuum. ▲, low chlorine content in air. △, low chlorine content in vacuum. **▼** ▲, no break at time indicated.

Evidence relative to molecular weight degradation and/or cross linking caused by high-energy radiation was obtained by zero-strength-time (ZST) measurements. No data were secured for PTFE. For PCTFE the molecular weight data derived from ZST-molecular weight correlations [11, 13, 14] are shown in table 8 and in figure 1. Correlations are not available for the other polymers, and the plots are of log ZST, which in general should have a linear relationship with molecular weight [11, 13]. The ZST data for TFE-HFP copolymer are given in table 9 and figure 2; for HFP-YF copolymer in table 9 and figure 3; and for two grades of CTFE-VF copolymer in table 9 and figure 4. All irradiations were made at 20±2 °C. The ZST data for PCTFE show a good linear relationship between the reciprocal of the number-average molecular weight, 1/M*_n_* and the radiation dose, indicating a rather constant *G*(scissions) of 0.67, i.e., nearly 0.67 scissions per 100 electron-volts of energy absorbed from the radiation, independent of the presence of air. The scatter of individual determinations was of the order of 5 percent, in agreement with earlier experience [11, 13]. The *G*(scissions) is low compared to values for typical degrading polymers such as polymethyl methacrylate (PMMA) and polyisobutylene (PIB), for which *G*(scissions) are 1.6 and 5, respectively [15, 16]. The insensitivity to air is surprising in view of the great sensitivity of PTFE (in tensile tests [8]) and of the TFE-HFP copolymer (fig. 2) and the definite air sensitivity of the copolymer HFP-YF (table 9).

Excepting possibly PTFE (for which ZST was not studied here) PCTFE was the only polymer in the group to show only scission. All the others, including even the pure fluorocarbon TFE-HFP copolymer, showed a period of rising ZST in the region up to 1–10×10^20^ ev/g, after which degradation usually began to dominate, as indicated by a gradual lowering of ZST. The approach to the maximum ZST is not a convenient measure of gel-point phenomena, as prohibitively high ZST’s, complicated by attendant thermal degradation of the sample, are reached without any sharp break in the rising ZST curve. From the theory of crosslinked networks it appears unlikely that a sudden break in ZST should be expected. In the cross linking systems, ZST test specimens subsequent to the maximum often showed a transverse fracture rather than a fine drawn-out thread, and the scatter of individual determinations then became great, specimens within a small tube showing deviations of 50 percent. This phenomenon has been observed before [17], although not explicitly associated with cross linking. Some samples at high dose (table 9), despite a relatively high ZST, were quite brittle and required careful handling. Among the CTFE-VF elastomers, the relative rate of degradation was evidently much greater in the material of high chlorine content. If the difference in chemical analysis is due solely to monomer ratio in the copolymer, the change from about 30 to 44 mole-percent CTFE is accompanied by a drastic increase in ease of scission. Samples of irradiated PTrFE and PTFS, although not examined by ZST, appeared to cross link, as evidenced by swelling and insolubility in pyridine and methyl ethyl ketone.

The results on volatile products are subject to serious scatter; in some cases a given product is reported less abundant after a post-irradiation heating than before it, and inconsistencies approaching twofold appear for products of low yield, for example, CF_4_ in table 3. Heating after irradiation had little demonstrable effect on yields of volatiles; however, a few products of higher molecular weight, absent before heating, appeared in trace amounts afterward, for example, C_4_F_8_ in table 3.

A major product was usually SiF_4_; however, PCTFE and the copolymer TFE-HFP yielded none. In the copolymer the absence of SiF_4_ may have been due to restricted diffusion of F atoms or other fragments from the polymer sample, which was in the form of 2-mm beads. The SiF_4_ was accompanied by CO_2_ of uncertain origin; CO may also have been present but was indistinguishable from small contaminations by atmospheric N_2_ during analysis. Possible sources of the CO_2_ are from the reactions of fluorocarbon radicals or unstable molecules with the glass walls of the vessel; carboxylic end groups in the polymer; or attack on radicals or double bonds by O_2_ indirectly produced from container walls.

**Figure:**
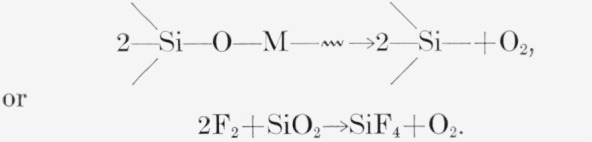


or

$$2F_{2}+{\text{SiO}}_{2}\to {\text{SiF}}_{4}+O_{2}.$$

Since the use of loose metal-foil wrappers did not appreciably diminish the yields of SiF_4_ and CO_2_ (table 3), the formation of these products from radicals appears unlikely, as the species responsible has long enough life to diffuse through folds of the wrapper.

There is some uncertainty about the origin of H_2_ from hydrogen-containing polymers. Possible sources are direct production from the polymer by an atomic or molecular mechanism, or reaction of initially produced HF with metal-foil wrappers. The reaction of HF with dry metal surfaces seems unlikely, however, and possibly all the H_2_ recorded arises from the polymer.

In addition to the mass spectrometric determinations, HCl and a trace of Cl_2_ were identified qualitatively from one tube of irradiated CTFE-VF copolymer; SiF_4_ and H_2_ may also have been present, and the pressure of more than one atmosphere would correspond to a total gas *G*-value in the neighborhood of 2 to 4.

No polymer yielded monomer as an important product. Some confusion was possible in the mass spectra of products from PTFE and PTrFE, where peaks were identified corresponding to the monomer mass numbers of 100 and 82, respectively; but in these instances the remainder of the mass spectrum was incorrect for the monomer, and the peaks in question were due to other products. Very small amounts of C_2_F_4_ corresponding to *G*=0.006 appeared from PTFE irradiated to 68.9×10^20^ ev/g and then heated at 400 °C for 20 min (table 3); and C_3_F_6_ equivalent to *G*=0.005 was present in irradiated TFE-HFP copolymer heated to 280 °C (table 4). A little C_3_F_6_ was also observed from the HFP-VF copolymer (table 7).

Both PTFE and PCTFE yielded numerous unidentified halocarbon products; however, the total of all volatile products was small, as the values of *G* (total gas) indicate (tables 3 and 5). In irradiated PTFE some material sublimes at 300 °C, producing a faint white ring, suggesting the presence of some products of intermediate molecular weight.

Any trend in the production of CF_4_ from PTFE was obscured by the large scatter; the *G*-values were in the range 0.004 to 0.009 for doses up to 1.84×10^22^ ev/g, which are lower on the average than Charlesby’s values [18] and do not seem to fit his dose-dependence formula requiring a regular linear increase from *G*=0 initially to *G*=0.050 at 1×10^21^ ev/g. At very high doses agreement might improve. The observations of Charlesby do not exclude some C_2_F_6_ and may have an uncertainty of nearly twofold based on uncertainties in the dosimetry.

**Figure 5:**
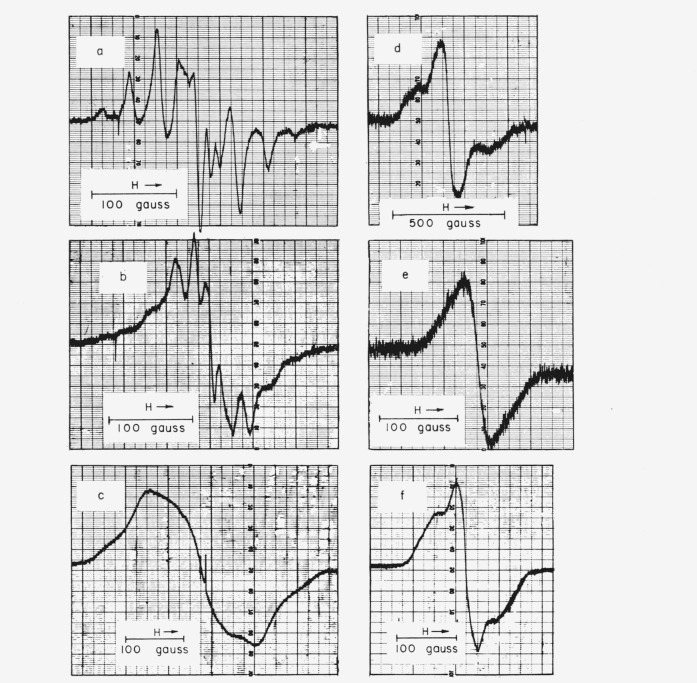
Electron spin resonance spectra of irradiated fluorocarbon polymers. a. Polytetrafluoroethylene. b. Tetrafluoroethylene-hexafluoropropylene copolymer. c. Polytrifluoroethylene. d. Polychlorotrifluoroethylene. e. Poly-2,3,4,5,6-pentafluorostyrene. f. Poly-α,β,β-trifluorostyrene.

**Figure 6:**
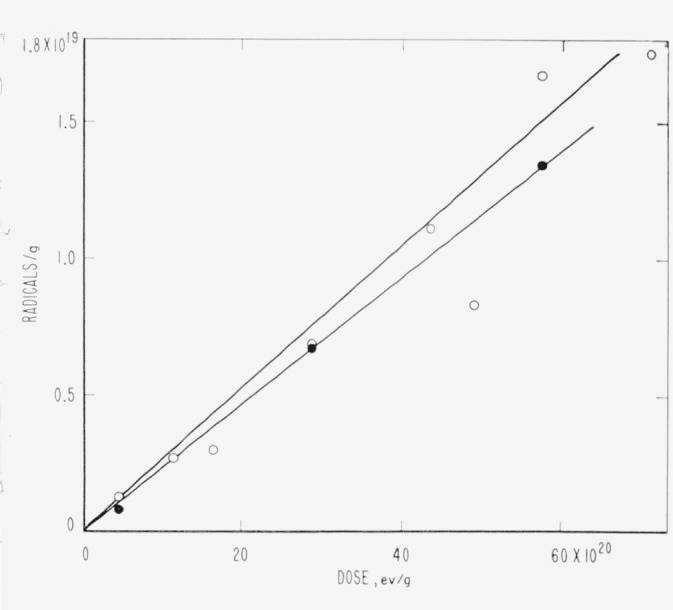
Accumulation of radicals in irradiated polytetrafluoroethylene. ○, stored 1 to 10 hr at 77 °K, error ±50 percent. ●, stored 5 mo at 300 °K, error ±20 percent.

**Figure 7:**
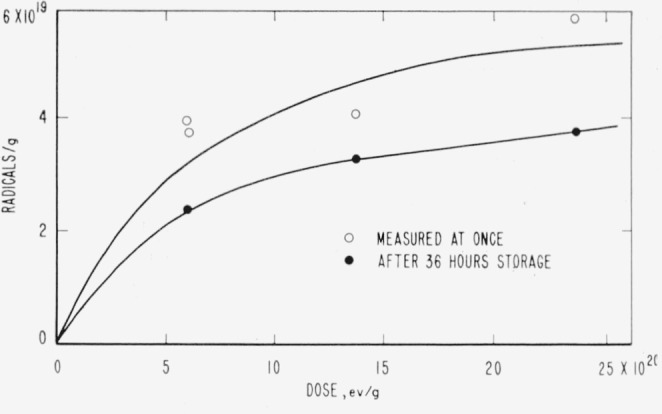
Accumulation of free radicals in irradiated copolymer tetrafluoroethylene-hexafluoropropylene. ○, stored 1 to 5 hr at 77 °K. **●**, stored 36 hr at 300 °K.

ESR spectra for PTFE, TFE-HFP copolymer, PCTFE, PTrFE, PTFS, and PPFS are shown in figure 5, and data on yields and spacings are given in table 10. The rubbery VF copolymers had no ESR spectrum, at least when irradiated at room temperature. The spectra of the styrenes and of PTFE are shown only for comparison; the styrenes have been discussed elsewhere [19], and the ESR spectrum of PTFE has been investigated extensively by other workers [20–23], the more recent of whom are in essential agreement, except as to yield. All the spectra are quite broad. PTFE alone has a sharply resolved hyperfine structure (*hfs*), but the PTFE-HFP copolymer is similar in many respects, the main differences being associated with the poorer resolution. Figures 6 and 7 show the accumulation and decay of radicals in irradiated TFE-HFP copolymer. Single irradiations of PTFE were made at 77 °K and 4.2 °K; at 77 °K the *hfs* was lost by broadening, as mentioned by Voevodskii [21]; at 4.2 °K the main spectrum was distorted by relaxation effects and two hydrogen atom lines appeared, the origin of which could have been either in the container or in hydrogen-containing impurities such as soap.

## 4. ESR Spectra

The ESR spectra clearly show the presence of free-radical species, but, of course, yield no information as to their role in the mechanism of the chemical changes. The radical concentrations are known approximately, and the *hfs* gives clues to the identity; however, most of the identifications in polymers are tentative because of the possibility of unresolved or faint *hf* components.

For the radicals in irradiated PTFE all recent workers find an ESR spectrum of 10 lines (rarely 11) covering 225 gauss [20–22], in essential agreement with figure 5a. Earlier reported experiments indicated three lines [24], eight lines [25], or else no spectrum until air had been admitted [26]. The spectrum is very reasonably attributed to the secondary radical ~ CF_2_ĊFCF_2_ ~, where the *hf* interaction is with one *α* and four equal *β* fluorines [20]. There is no indication of any primary radicals 〰 ĊF_2_, which would be intermediates in chain scission. A possible explanation is that pairs of primary radicals, if formed by C—C scission, are held in a cage until they recombine, while fluorine atoms that split off during formation of secondary radicals can diffuse away more easily because of their small size. The resolution of *hfs* is very good for a polymer at room temperature, but reversibly broadened out at 77 °K; the broadening is no doubt caused by the loss of motional freedom on cooling, in agreement with NMR studies [27]. The ~ CF_2_ĊFCF_2_ ~ radicals need not undergo scission and may form cross links.

The radicals combine readily with oxygen and several other agents, as might be expected of a free radical [20]. The peroxy radical has a much narrower spectrum than the parent fluorocarbon radical. There is some recent evidence that the combination with oxygen is partially reversed by heating, and that two kinds of peroxy radicals may exist [22]. The yields of radicals, *G*(R)=0.16 to 0.19 for PTFE and *G*(R) = 1.1 for HFP copolymer, are comparable with the yields of volatile products, and the decay is quite slow (figs. 6 and 7). For PTFE the buildup of concentration was linear with dose to 64×10^20^ ev/g at least. The *G*-value, growth curve, and decay rate conflict somewhat with Watanabe’s results from deuteron bombardment [23], where the initial *G*-value appears to be as low as 0.05 and the leveling off of radical concentration at higher doses fits a first-order decay constant of 2.8×10^−3^ sec^−1^. It seems likely that Watanabe’s low *G*(R) may be due to a high local temperature and linear energy transfer associated with deuteron beams, and that the large first-order decay constant applies only while the irradiation is in progress.

Watanabe has suggested two mechanisms for a first-order disappearance: $〰{\text{CF}}_{2}\dot{C}F_{2}+\dot{C}F_{2}{\text{CF}}_{2}〰(\text{in}\;\text{cage})\to 〰{\text{CF}}_{2}{\text{CF}}_{2}{\text{CF}}_{2}{\text{CF}}_{2}〰\text{and}〰{\text{CF}}_{2}\dot{C}F_{2}〰\to 〰\text{CF}=\text{CF}〰+\dot{F}$. In the TFE-HFP copolymer the growth curve levels off (fig. 7), and a moderately rapid decay occurs initially. Both the more rapid decay and the greater diffuseness of *hfs*, compared with PTFE, may be attributed to lower crystallinity; some of the differences may also be due to the superposition of several radical spectra; for example,

**Figure:**
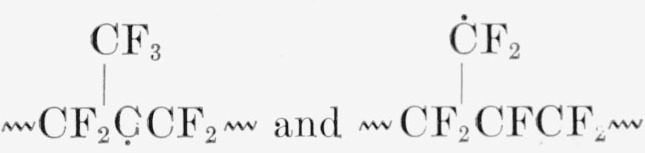


In irradiated PCTFE the initial *G*(R) of about 1.0 is comparable with the estimated *G*(scissions) = 0.67 and much less than the *G*(F^−^) and *G*(Cl^−^) [5] of polymer irradiated in aqueous alkali and air. Previous studies indicated either no detectable radicals [26] or an *hfs* of several unresolved lines [28] if irradiated *in vacuum*, and a *G*(R) of 0.5 [26] if exposed to air during or after irradiation. The three-peak structure here is too diffuse to support conjectures as to identity. The most favored radical energetically should be 〰 CF_2_ĊFCF_2_CFCl 〰 formed by removal of chlorine; it should have the same *hfs* as the radical from PTFE. In the 〰CF_2_ĊFCF_2_〰 radical of PTFE, as analyzed by Rex-road and Gordy [20], the *α* fluorine interaction is 92 gauss and the *β* interaction 33 gauss. A radical 〰CFClĊF_2_ would have the requisite two *α* fluorines to produce the 3-peak structure with 100-gauss separation; the smaller splittings by the *β* fluorine could be obscured. Such a radical could be formed by a primary C—C scission or also by the breaking of an initial secondary radical formed by C—F splitting.

$$\begin{array}{l} 〰CF_{2}\text{CFCl}\dot{C}\text{FCFClC}F_{2}\text{CFCl}〰\to \\ 〰CF_{2}\text{CFClCF}=\text{CFCl}+\dot{C}F_{2}\text{CFCl}〰 \end{array}$$

The initial radical shown in this equation, although requiring more energy for formation, could be favored by greater mobility of the F atom removed. The diffuse spectrum actually found is compatible with the simultaneous existence of several kinds of radicals.

In the other irradiated polymers, as in PCTFE, the radical spectra are too diffuse to be very helpful for identification; the yields are moderately large in PTrFE and PTFS but very small in PPFS, suggesting stabilization against bond rupture by the pentafluorophenyl ring.

Besides the evidence for radicals, there are, in the literature, indications of the transient existence of both charged species and excited states. A temporary increase in electrical conductivity occurs during the irradiation of PTFE [7,29,30] and PCTFE [31], and persists for hours afterward, disappearing more rapidly at higher temperatures. A very weak phosphorescence also appears upon warming PTFE irradiated in vacuum at 77 °K [29]. The chemical importance of the species concerned is doubtful, and no definite speculations have been made regarding the emission process, nor is anything known of the identity, mobility, and concentration of the current-carrying species. Speculations have been made, however, concerning the possible role of ions in fluorocarbon radiation chemistry [32]. In irradiated PTFE the identification of the radicals as 〰CF_2_ĊFCF_2_〰 is reasonably sure, and much of the known radiation behavior of the polymer can be explained in terms of them.

## 5. Products of Irradiation

Recent experiments on the irradiation of small fluorocarbon molecules do not indicate abnormally high G-values for products. The rapid polymerization of TFE and of CTFE by *γ*-rays may seem an exception, but in view of the high molecular weight of the polymer the *G*-value for initiation is not necessarily high. Gamma rays affect C_3_F_6_ remarkably slowly, and high polymer is not formed [33]. When perfluoroheptane is irradiated in vacuo in dry aluminum containers, scission products are present in small amounts only, no corrosion or inorganic fluoride is seen, and the irradiated material contains coupling products [9, 10]. In nickel tubes with glass capillary ends, small amounts of SiF_4_ are seen also [10]. A few of the *G*-values of products from C_7_F_16_ are given in table 11. Low *G*-values of products were found in CF_4_ mixtures [32, 34]. From CF_4_ mixed with C_6_H_6_, the *G*-values of C_6_H_5_F and C_6_H_5_CF_3_ together amounted to about 1.

The polymers studied fall into two distinct groups: (a) the hydrogen-containing polymers, which evolve HF or HCl and cross link rapidly, and (b) the pure halocarbon polymers, which cannot evolve HF or HCl and cross link more slowly, if at all. A special class may be constituted by the silicones containing perfluoroalkyl groups, which the literature reports to be quite sensitive to radiation [35].

Haszeldine has prepared copolymers of CF_3_NO and C_2_F_4_ [36] and polymers of CF_2_ = CF–NO [37], which show promise as elastomers. He has also prepared an unsaturated thermally stable polymer of structure –CF=N– [37]. Although radiation stability of the first polymer would presumably be low, no data are available on these polymers or their analogs.

Fluoroaromatic polymers of several types have been made in small quantities. Representative types include PTFS (fluorocarbon main chain and hydrocarbon ring), PPFS (hydrocarbon chain an fluorocarbon ring), and polyperfluoropolyphenyl (perfluoroaromatic rings linked directly). The thermal stability of the latter two polymers appears to be good [38,39]. Further aromatic systems such as perfluorophenylene ethers may be possible. Irradiation of the prototype molecule C_6_F_6_ resulted in coupling to form polymer as the main reaction, and produced almost no inorganic fluoride or small molecules [10]. The triazine polymers developed by H. C. Brown [40] have a quasi-aromatic ring structure, and some examples are thermally stable [38], but no radiation data are known. Among the pure halocarbon polymers, PTFE offers special problems and will be considered later.

### 5.1. PCTFE

For PCTFE the radiation resistance in terms of physical properties was rated low, similar to PTFE [3,5]. There were high yields of ionic products from irradiations in dilute alkali and air; *G*(F^−^) = *G*(Cl^−^) = 3.5, approximately [5]. In the present study an uncomplicated scission process seems established, with constant *G* (scissions) of 0.67 (see fig. 1). This value is not high compared to those of such polymers as PMMA. The absence of SiF_4_ from irradiated PCTFE (table 8) is curious and could be due to the easier breaking of C—Cl bonds. The low yields of any volatile products in vacuum irradiation contrast with the very high and equal yields of Cl^−^ and F^−^ for irradiations in the presence of water and oxygen [5]. A smaller discrepancy also exists between F^−^ yields from PTFE in aqueous and evacuated systems [4,41,42] (see table 12).

For PCTFE in vacuum, possible reactions are:

**Figure:**
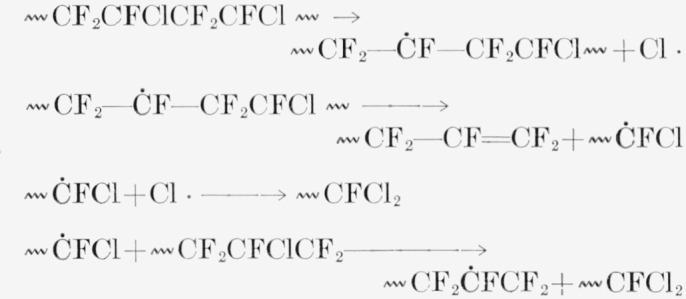

If air and water are present, the radicals can be converted to peroxide radicals and ultimately hydrolyzed:

**Figure:**
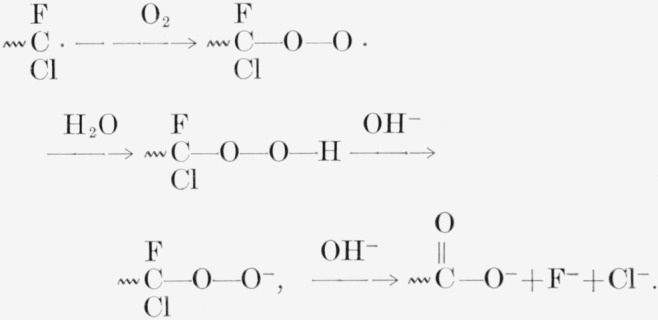


In PTFE irradiated in air the reported development of appreciable water absorption [4] may be due likewise to the formation of carboxylic acid groups. The curious insensitivity of the PCTFE molecular weight to the presence of oxygen during irradiation may be due to the relative stability of peroxide radicals of this form, at least in the absence of water and alkali, or to the fact that the molecular weight drop is already occurring so rapidly in the absence of air.

### 5.2. Hydrogen-Containing Polymers

Polymers containing hydrogen have previously been found to undergo the changes associated with cross linking: vulcanization at 10 Mr or less [43, 44, 45], followed by a slow loss in elongation [46]. Copolymers of HFP and VF have marginal utility at 100 Mr according to evaluation studies. Similar results are shown for the PCTFE-VF copolymer and for perfluorodihydroacrylate polymers. The specific data quoted by Harrington [35, 46] at 100 Mr indicate a loss in tensile no greater than 36 percent for any of these three polymers, but about 85 percent loss of elongation for the acrylate and the HFP-VF copolymer.

In most of these hydrogen-containing polymers the evolution of hydrogen fluoride was observed qualitatively. Small molecules containing hydrogen as well as fluorocarbon groups have hardly been studied at all under irradiation; however, mixtures of fluorocarbons with hydrocarbons evolve hydrogen fluoride in large amounts [10], and the evolution of hydrogen fluoride is also reasonably expected if the hydrogen and fluorine are in the same molecule, as in VF. For fluorine-containing polymers the evolution of the highly stable molecule HF should be associated with cross linking as H_2_ is for polyethylene.

The predominance of cross linking is shown by the trend of the ZST curves, figures 3 and 4. The associated high *G*(HF), (tables 6 and 7) and the implicit high *G*(HCl) are not surprising. Despite the well-developed cross linking, scission ultimately dominates. The greater tendency to scission (or smaller cross linking tendency) of the CTFE-VF copolymers is evident, especially for the copolymer of high Cl content. The HFP copolymer evolves a certain amount of C_3_F_6_, CF_4_, and H_2_, despite the competition of cross linking and HF evolution processes. For this class of polymers, especially the HFP-VF copolymer, it is interesting to note that long retention of useful properties [46] is not forbidden by a high rate of evolution of corrosive products.

In PTrFE the production of CF_3_H is surprising. A possible but unconvincing route to it could exist in a mechanism similar to those quoted for CF_4_ from PTFE [41, 42, 47]. For PTFE, either of the following reactions gives CF_4_:

**Figure:**
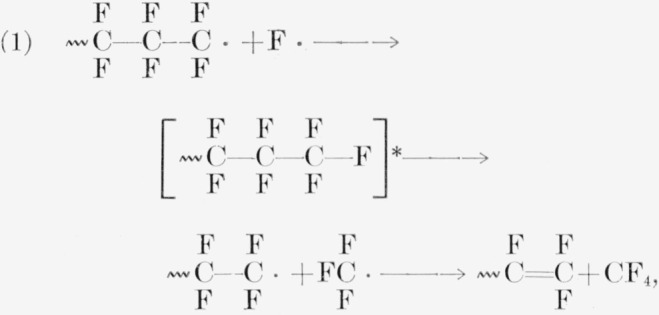

**Figure:**
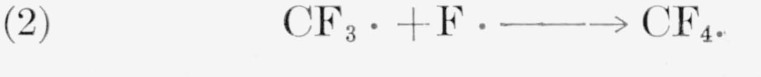

For PTrFE two of the three following reactions produce CF_3_H:

**Figure:**
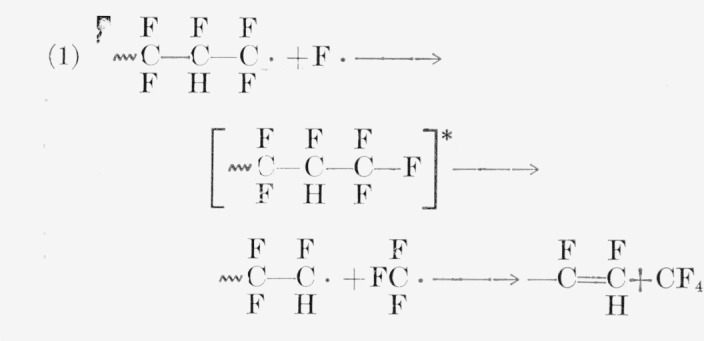

**Figure:**
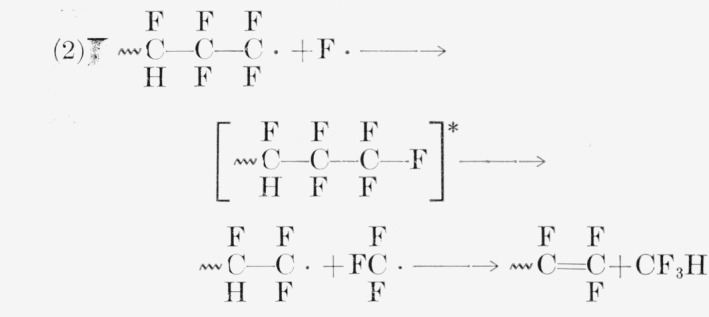

**Figure:**
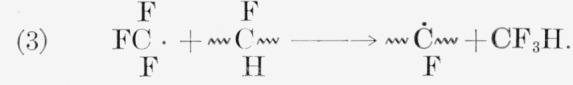

Both the above mechanisms for CF_3_H and the high yields of SiF_4_ (via HF) are favored by the probable frequent occurrence of head-to-head bonds 〰CF_2_—CFH—CFH—CF_2_—CF_2_—CFH, 〰 a consequence of the nearly equal reactivity of the monomer for radical addition at either carbon atom [48].

### 5.3. PTFE

The radiation stability of PTFE remains an unsettled problem in several respects, although PTFE has been investigated for the longest time. Contributing factors to this situation are the extreme sensitivity to the presence of oxygen during irradiation [8] and the difficulty of measuring the properties related to molecular weight [17,49,50]. The tensile strength of PTFE film irradiated in air drops to zero after a few megaroentgens exposure, whereas with irradiation in vacuum there is an indefinitely long plateau at 50 percent of the original strength. Irradiation of thicker specimens, or irradiation in low vacuum, must show intermediate grades of behavior, depending upon the relation of dose rates, diffusion rates, and oxygen supply. The copolymer of PTFE and HFP, studied by ZST measurements, is also highly sensitive to irradiation atmosphere (table 9, fig. 2), whereas PCTFE is not (table 8, fig. 1).

The course of molecular-weight degradation and cross linking cannot be followed readily by the usual solution methods, as PTFE is insoluble except in special solvents at 320 °C and higher; observations of the usual properties including intrinsic viscosity, light scattering, osmotic pressure, and swelling in solvents have rarely been achieved. A few special molecular-weight methods have been calibrated by reference to end-group analysis as an ultimate standard. The reference standard involves assumptions about polymerization mechanism. Melt viscosity methods are available, but the most consistent methods at present appear to be based upon the density or crystallinity, following a carefully programmed annealing period [51]. In PTFE irradiation, some use has been made of crystallinity and density [41, 42, 52], but not as explicit measures of molecular weight. In the absence of more significant measurements much work has been done with mechanical properties, including impact strength [2], tensile strength and elongation [28, 46], and creep rate [50, 53]. The creep rate may have been rather closely connected with melt viscosity, which has been correlated with molecular weight. ZST measurements at 350 °C have been applied and correlated with molecular weight, but the behavior is not typical, and the results scatter badly [17, 52, 53]. The ZST measurement is more easily applied to the copolymer of TFE with HFP (see Experimental Procedures).

An undesirable feature of tensile strength measurements is that the property is generally sensitive to molecular weight in an intermediate range only, being zero at low molecular weights and reaching an upper limit at high molecular weights [54].

The observed changes of mechanical properties are, (1) a very early increase in impact strength at 3×10^20^ ev/g [2], (2) a loss of most elongation somewhere in the range 0.5–5×10^20^ ev/g [2,46], (3) a loss of tensile strength, which may occur early or not be important until past 30×10^20^ ev/g [8,46], and finally (4) a disintegration of large pieces beginning around 300×10^20^ ev/g [18]. Thin pieces are more resistant to disintegration. The above observations apply to irradiations in which oxygen was usually not of major importance because of evacuation or of sample thickness. Irradiations conducted in air at room temperature caused a very rapid drop in ZST, melt viscosity, and activation energy for flow, and an increase in density and crystallinity [52,53,55].

To summarize, for PTFE specimens irradiated in evacuated containers there are many empirical data on properties but there is no information closely related to molecular weight, whereas for specimens irradiated in air the systematic data related to molecular weight indicate a very rapid degradation, important at doses as low as 0.2×10^20^ ev/g. For the related HFP copolymer the present ZST data are compatible with cross linking and very slow degradation in vacuum, and with very rapid degradation in air (fig. 2).

Volatile and ionic products sometimes show a dependence upon thickness [4,18] or upon storage after irradiation [4], which is attributed to slow diffusion. In the present study these effects were small because of the powdered form of the sample and the long storage before analysis. The initial *G*-value for evolution of F-in aqueous alkali and air was near 0.6 or 1.7 in different studies. A weight loss proportional to the square of the radiation dose was found by Charlesby [18] when diffusion effects were eliminated. If the weight loss was principally CF_4_, the *G*(CF_4_) should increase proportionally with dose. The identification of weight loss as CF_4_ was only tentative.

In the present study CF_4_ was not an especially abundant product, and 6r(CF_4_) did not increase notably with dose. The Charlesby relation may possibly hold for CF_4_ at very high doses and higher temperatures. The CF_4_ from the HFP copolymers (table 4) may indicate a tendency to break at branch points. No monomer was found after irradiation, and only a very little was found after heating irradiated polymer (table 3), in contrast with the reported behavior of poly (methyl methacrylate) [56]. The irradiation of PTFE in a furnace, however, is stated to yield monomer rapidly if irradiation is done above 325 °C [57]. Among incidental chemical or physical observations are an increased water absorption when irradiated in air [4], a change in X-ray spacing parameters [41], and permanganate titrations and infrared spectra suggestive of two kinds of double bonds. The double bonds and ionic fluoride mentioned earlier are not apparent in the irradiation of the chemically analogous perfluoroheptane. As mentioned earlier the ESR spectrum indicates the presence of a secondary radical 〰CF_2_ĊFCF_2_〰, which is quite stable in vacuum but reacts rapidly with oxygen.

The pertinent radiation yields from new and old work are listed in table 13. Earlier discussions of PTFE regarded the polymer as degrading exclusively, as much of the qualitative evidence seemed to imply. Thermochemical estimates of the several bond energies, F—F = 37 kcal/mole, C—C = 83 kcal/mole, C—F = 105 kcal/mole [58], made cross linking, with elimination of F_2_, appear especially unfavorable energetically so that C—C scission would dominate in competition. The identification of radicals 〰 CF_2_ĊF—CF_2_〰 shows that C—F splitting actually occurs; therefore, not energetics but cage effects and relative diffusion rates are the dominant factors, and C—C scission is no longer the only allowed process. From the parabolically increasing yields of gas (regarded as CF4) and a picture of random C—C scission, Charlesby had arrived at a *G*(C—C scission) =2, of the same order as that found in polyethylene. Nishioka’s melt viscosity data led to the much higher *G*(C—C scissions) ≈ 10 for degradation in air.

Detailed chemical steps suggested were the following:

**Figure:**
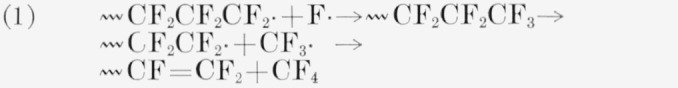

[41,42,47]

**Figure:**
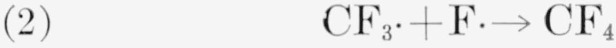

[47]

**Figure:**
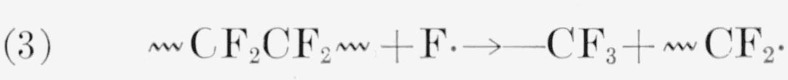

[59]

The steps have accounted for the double bonds that were found [41]. A secondary effect was the distortion of crystal structure; since double bonds are shorter than single bonds and the angles are different, great strain is expected in the compound helix structure [41], and a disturbance of spacing was apparently found. The tendency to disintegrate was attributed either to the crystal strains [41] or to the pressure of relatively nondiffusing CF_4_ accumulated in the solid [18,59].

Nothing more has been learned about the mechanisms of breakage. Indirectly, the analysis of volatile products from PTFE and of all products from the liquid *n–*C_7_F_16_ [9,10] do not suggest important amounts of olefins. The superior retention of tensile strength in thin specimens of PTFE [8,18,60] may suggest that gas inclusions rather than crystal stresses cause the observed failures of thicker specimens.

The actual extent of molecular weight degradation is unsettled, largely because of the difficulties of measurement. The relatively careful measurements by Nishioka et al., [53] based largely on melt viscosity, were made upon samples irradiated in air, and the huge *G* (scission) value of 10 deduced from those measurements can apply only to the process in air. A more rigorous recalculation in terms of the best available molecular-weight relationships would be of interest. The tensile-strength measurements reported from this laboratory [47] suggest a very slow or zero rate of scission in vacuum. A few test data indicate a relatively slow loss of tensile strength but drastic loss of elongation [46]. These irradiations may have been performed in relatively good vacuum. However, the most favorable previous results indicate loss of most mechanical strength at a dose of about 0.5×10^22^ ev/g. A certain amount of cross linking is indicated by the ZST data for TFE-HFP copolymer, and possibly by the reported initial increase in impact strength of PTFE [2]. As has been mentioned, the free radical species 〰CF_2_ĊFCF_2_〰, which should be able to cross link, is the only one identified and is prominent.

Thermochemical considerations indicate very slight possibility for reaction by these radicals at ordinary temperatures, except possibly cross linking. Combination of small fluorocarbon radicals occurs readily enough, although perhaps more slowly than the normal hydrocarbon rate [61]; (for other references see [10]).

Abstraction and disproportionation reactions have not been reported for fluorocarbon radicals and chain compounds up to high temperatures, and abstraction of F even by hydrogen atoms involves 17 kcal/mole or more (for references see [10]). The secondary radical could split at high temperature into an olefin and a primary radical, which could then split off monomer.

**Figure:**
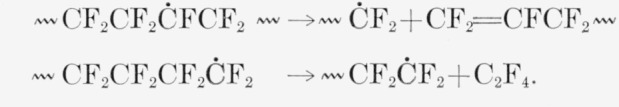


The last reaction is the reverse propagation step of polymer pyrolysis, for which the activation energy is necessarily greater than 46 kcal/mole, which is equivalent to the heat of polymerization [62]. Reverse propagation would occur to a negligible extent at room temperature, but one might have expected the formation of monomer at high temperatures, as is the case with PMMA [56]. Actually upon heating from 20 °C to 400 °C, a sample estimated to contain 3×10^18^ radicals ((*G*_R_/100). $D.\text{W}=\left(\begin{array}{c} 0.2\\ 100 \end{array}\right)\times 6.89\times 10^{20}\;\text{ev}/\text{g}\times 0.2\;g$) (tables 13 and 3) evolved only 8.4×10^16^ molecules of C_2_F_4_ at 0.03 molecule per radical. The oxygenated radicals produced by exposure to air also give rise to very little decomposition of any kind when heated to 310 °C in vacuo (table 3, last column). Since the radicals disappear rapidly at 320 °C, most of them probably combine before the samples reach the temperature needed for rapid depropagation. If radicals could be produced continuously at 400 °C or so by irradiating a heated sample, a significant rate of depropagation might be found. Rapid depropagation evidently occurred in a sample of PTFE irradiated at a nominal temperature of 330 to 350 °C [57]. The weight loss of a PTFE sample irradiated to a dose of 12.7×10^21^ ev/ml, at a dose rate of 42×10^18^ ev/ml-sec, jumped from a level near 0.5 percent below 300° to 50 percent at 330 to 350 °C.

With more closely controlled temperatures, and observation of radicals under identical conditions, the constants of the depropagation process could be isolated. The data of Taubman et al., [57] indicate a *G*(C_2_F_4_) of perhaps 30 molecules per 100 ev at 330 to 350 °C. If we assume that the rate of formation of depropagating radicals is given by the *G*-value of secondary radicals observed at room temperature, the data imply that each radical formed at 330 to 350 °C evolves on the average 150 molecules of C_2_F_4_ during its lifetime.

## 6. Conclusions

When fluorocarbon polymers are irradiated in vacuum, the observed yields of products from splitting the C—F and C—C bonds are often less than those from the C—H and C—C bonds in hydrocarbon polymers. The accompanying corrosion may, of course, be more serious. Cross linking, followed by degradation, occurs in polymers containing both F and H, and also in the pure fluorocarbon copolymer TFE-HFP. Chain scission alone occurs in PCTFE. Probably both processes occur in PTFE, with little net change in tensile strength. For both PTFE and its HFP copolymer the radiation behavior is very sensitive to the presence of oxygen. The radiation of PCTFE is insensitive to oxygen with respect to molecular weight degradation, which is moderately rapid in any event, but very sensitive with respect to loss of F and Cl in the presence of air, water, and alkali.

In many of the irradiated polymers free radicals can be observed, sometimes at *G*-values as large as 1. In the only perfluoroaromatic ring polymer studied, PPFS, the *G*(R) was very low, similar to that in polystyrene, suggesting that perfluoroaromatic polymers would have superior radiation resistance.

There are many unsolved problems in the radiation chemistry of PTFE, particularly in regard to the true rates of scission and cross linking in vacuum, the possibility of predominant cross linking, and the ultimate fate and kinetic importance of the observed free radicals.

## Figures and Tables

**Table 1:** Copolymer compositions

Copolymer	C	H	C	F	Monomer
					
	*wt %*	*wt %*	*wt %*	*wt %*	*Mole %*
PTFE-HFP	…………	…………	…………	…………	11%HFP
HFP-VF					
found	32.2	2.4	0	64.4	18%HFP
calc	32.8	2.1	0	65.1	…………
CTFE-VF (high Cl)					
found	27.0	1.3	15.5	52.5	44%CTFE
calc	27.6	1.3	17.9	53.3	…………
CTFE-VF (low Cl)					
found	29.6	1.7	11.3	56.4	30%CTFE
calc	30.1	1.8	13.4	54.8	…………

**Table 2:** Molding and ZST conditions^a^

Polymer	Mold temperature	Time of heat	Time of press.	ZST temp
				
	*°C*	*min*	*min*	*°C*
PCTFE	260	4	2	250
TFE-HFP	………	………	………	280
CTFE-VF (high Cl)	177	5	15	214
CTFE-VF (low Cl)	163	5	15	211
HFP-VF	127	3	10	120

a Spacers 0.075 in.; test strip 2 in. long, 0.187 in. wide, 0.062 in. thick, except TFE-HFP copolymer, 0.060 and 0.040 in. thick; notch 0.047 in.

**Table 3:** G-values of volatile products from polytetrafluoroethylene^a^

Dose, (ev/g)×10^−20^	34.2	34.2	68.9	68.9	184	184	184
							
Container and treatment^b^	N	NP	N	NP	G	GQ	GR
							
SiF_4_	0.16	0.12	0.16	0.31	0.15	0.21	0.005
CO_2_	.12	.13	.11	.11	.06	.12	.02
CF_4_	.004	.009	.007	.007	.009	.005	.006
C_2_F_6_	0	0	0	0	tr.	.007	0
C_2_F_4_	0	0	0	0.006	0	0	0
C_4_F_8_	0	0	0	.004	0	0.005	0
Total gas	0.32	0.31	0.30	.30	………	………	………

a In powder form.

b Symbols:

G—glass tube, no liner;

N—nickel foil wrapper;

P—postheated 400 °C, 20 min:

Q—postheated 300 °C, 30 min;

R—further increment produced by air and heat; irradiated sample was opened to air, re-evacuated, then heated at 310 to 320°, 15 min.

**Table 4:** G-values of volatile products from a tetrafluoroethylene-hexafluoropropylene copolymer^a^

Dose, (ev/g)×10^−20^	9.0	34.4	34.4^b^	69.3^b^
				
SiF_4_	0	0	0	0.0005
CO_2_	0 022	0.008	0.026	.011
CF_4_	.063	.085	.089	.113
C_3_F_6_	0	0	.005	.001
C_4_F_8_	0	0	.018	.022
C_5_F_12_	0	0	.006	.011
Total	0.102	0.107	.156	.169

a Beads of polymer, glass tubes, nickel foil liners.

b Heated after irradiation 280 °C, 15 min.

**Table 5:** G-values of volatile products from polychlorotrifluoroethylene^a^

Dose, (ev/g)×10^−20^	21.8	66.1	66.1^c^
Total gas^b^	0.11	>0.13	>0.14

a Glass tubes, silver foil wrapper.

b Mainly unidentified C, Cl, F compounds having up to 5 C and 2 Cl; no SiF_4_; no Cl_2_; little CO2.

c Heated after irradiation 250 °C, 15 min.

**Table 6:** G-values of volatile products from polytrifluoroethylene^a^,^c^

Dose, (ev/g)×10^−20^	12.2	39.5	67.3	67.3^b^
				
SiF_4_	0.668	0.767	0.864	0.94
CHF_3_	.018	.025	.016	.029
CO_2_	.092	.098	.113	.123
CO	.243	.147	.137	.14
H_2_	.028	.033	.120	.091

a In powder form, glass tubes, aluminum foil wrapper.

b Heated after irradiation 100 °C, 1 hr.

c All samples also showed unidentified fragments of mass 82, but different from CF_2_CFH.

**Table 7:** G-values of volatile products from a hexafluoropropylene-vinylidene fluoride copolymer^a^

Dose, (ev/g)×10^−20^	9.23	35.1	35.1^b^
			
SiF_4_	0.29	>0.30	>0.26
CO_2_	.091	>.011	>.007
H_2_	.27	>.17	>.11
CF_4_	.045	>.031	>.025
C3F6	.01	0	0
Total gas	.86	>0.54	>0.43

a Shreds of polymer, glass tubes, nickel foil liners.

b Heated after irradiation 100 °C, 30 min.

**Table 8:** ZST and molecular weight data of irradiated polychlorotrifluoroethylene^a^

Lot	Dose	ZST	M*_n_*b
			
	*ev/g*×10^−20^	*sec*	
A^c^	$\left\{ \;\begin{array}{l} 0\\ 0.198\\ .602\\ 1.54\\ 3.71 \end{array}\right.$	307	352,000
		250	314,000
		197	271,000
		153	217,000
		111	150,000
A^d^	$\left\{ \;\begin{array}{l} 0.198\\ .602\\ 1.54\\ 3.71 \end{array}\right.$	241	311,000
		215	286,000
		139	196,000
		108	144,000
B^c^	$\left\{ \;\begin{array}{l} 0\\ 0\\ 5.6\\ 5.6\\ 27.8\\ 72.3 \end{array}\right.$	347	373,000
		e935	…………
		90	137,000
		e146	…………
		e95	(f50,000)
		e70	(f<10,000)

a ZST measured at 250 °C on standard notched strip [12, 13] unless otherwise indicated.

b From correlation chart [11, 13, 14].

c Irradiated in vacuum.

d Irradiated in air.

e ZST on whole strip without notch.

f Long extrapolation from chart.

**Table 9:** ZST values of irradiated polymers

Polymer	Dose	ZST
		
	*ev/g×*10^−20^	*sec*
TFE-HFP^a^,^c^	0	256
	0.197	232
	.600	276
	1.54	243
	3.69	371
	5.52	448
	24.4	195
	70.6	d123
TFE-HFP^b^,^c^	5.57	218
	24.4	(e)
HFP-VF^a^,^f^	0	356
	0.202	749
	.384	3275
	g.384	4030
	.616	49000
	5.72	h>260000
	28.4	h>260000
	74.0	h>260000
HFP-VF^b^,^f^	0.202	523
	.384	1080
	.616	3639
CTFE-VF^a^,^i^	0	1001
(15.5% Cl)	5.69	172
	28.2	21.6
	73.6	8
CTFE-VF^b^,^i^	0.201	901
(15.5% Cl)	.612	5988
	3.77	3330
CTFE-VF^a^,^i^	0	960
(11.3% Cl)	5.72	h>216000
	28.4	723
	74.0	67.6
CTFE-VF^b^,^i^	0.202	h>6700
(11.3% Cl)	3.79	h>80000

a Irradiated in vacuum.

b Irradiated in air.

c ZST at 280±0.5 °C. Thickness 0.060 in., or 0.040 in. converted to 0.060 in. basis.

d Rather brittle.

e Friable; could not be handled.

f ZST at 120°±0.5 °C.

g Heated after irradiation at 100 °C for 0.5 hr.

h No break; abandoned at time indicated.

i ZST at 214°±1 °C.

**Table 10:** ESR data from irradiated fluorocarbon polymers

Polymer	Number of peaks	Spacings	Width overall	Yield, *G*(R)
				
		*Gauss*^a^	*Gauss*	*Radicals/100 ev*
PTFE^b^	c10 or 11	−96, −67, −40, −18, −13, (−3, +4) +13, +23, +42, +71, +97	220	0.15, 0.19
TFE-HFP^b^	11	−88, −67, −40, −20. −11, (−5, +2), +12, +22, +40, +67, +93	220	1.1
PCTFE^d^	3	−100, (−5, +5), +100	350	0.99
PTrFE^d^	e5	−171^e^, −94, (−29, +29)^e^, +94, +156^e^	425	.74
PTFS^d^	3	−52, (−18, +19) +51	220	.57
PPFS^d^	1	……….	140	.11

a Derivative peak locations; pair in parentheses due to single center component.

b Irradiated at 20 °C.

c Varies with orientation, identity and age.

d Irradiated at −80 °C; observed at 25 °C.

e Very weak shoulders.

**Table 11:** G-values of products from perfluoroheptane

Product	G	Reference
		
C_7_F_16_ (disappearance)	3.0	9
F	0	9
SiF_4_	0.17	10
CF_4_	.19	10
CF_4_	0	9
C_2_F_6_	0.08	10
<C_7_	~1	9
>C_7_	~2	9
~C_13_–C_15_	1	9
C_13_–C_15_	2	10

**Table 12:** G-values of products from polychlorotrifluoroethylene and polytetrafluoroethylene

Product	Condition	PCTFE	PTFE
			
Cl^−^	in aq. alkali	3.5 [5]	
Cl	in vac	0 (table 5)	
F^−^	in aq. alkali	3.5 [5]	2.0, 0.66 [4]
F	in vac (as SiF_4_)	0 (table 5)	0.48 to 0.82 (table 3)

**Table 13:** G-values of products from irradiation of polytetrafluoroethylene

Product	Conditions	G	Reference
			
CF_4_	Pile	0.005 to 0.05^a^	18
CF_4_	γ, vac; (30 to 84)×10^20^ev/g	.004 to .009	Table 3
SiF_4_	Pile	≤CF_4_	18
SiF_4_	γ, vac	0.12 to 0.16	Table 3
F^−^	Water, air	2.0	41
C=C	Water, air; KMnO_4_	~0.2	41
Scissions	Pile, vac	2	18
Do	Air	10	53
Radicals	Deuterons	0.05	50
Do	γ, vac	0.16 to 0.19	Table 10
Do	Vac., then air	Peroxy 0.2	26
Do	Irr. air	Peroxy 0.03	26

a Linear with dose, 0.05 at 10^21^ev/g.

## Footnotes

1 Figures in brackets indicate the literature references at the end of this paper.
